# Injury and not the pathogen is the primary cause of corm rot in *Crocus sativus* (saffron)

**DOI:** 10.3389/fpls.2023.1074185

**Published:** 2023-01-24

**Authors:** Ritika Mansotra, Tahir Ali, Nancy Bhagat, Jyoti Vakhlu

**Affiliations:** Metagenomic Laboratory, School of Biotechnology, University of Jammu, Jammu, India

**Keywords:** saffron fields, *Fusarium oxysporum*, fungal pathogens, injury, saffron corms

## Abstract

*Fusarium oxysporum* has been reported to be the most devastating pathogen of *Crocus sativus* L., a commercially significant crop that yields the saffron spice. However, most of the pathogen isolations have been done from the diseased tissue, mostly from rotten corms, but no study has been conducted on diseased saffron fields. To fill the knowledge gap, the current study was carried out with the intention of recording the diversity of cultivable fungus species from saffron fields and screening them for pathogenicity towards saffron. The three study locations in Jammu and Kashmir, Srinagar (Pampore), Kishtwar, and Ramban, yielded a total of 45 fungal isolates. The internal transcribed spacer (ITS) of rDNA was used for the molecular identification. ITS rDNA-based sequence analysis classified all the operational taxonomic units (OTUs) into two phyla—Ascomycota (88.88%) and Mucoromycota (11.11%). Moreover, *Fusarium* (57.77%), *Geotrichum* (17.77%), *Mucor* (11.11%), *Aspergillus* (4.44%), *Trichoderma* (4.44%), *Galactomyces* (2.22%), and *Colletotrichum* (2.22%) all had different total abundances at the genus level. It was discovered that the saffron fields in Srinagar have fewer varied fungal species than the other two selected sites. All of the fungal isolates isolated including *Fusarium solani*, *Aspergillus flavus*, *Trichoderma harzianum*, *Fusarium neocosmosporiellum*, and *Mucor circinelloides* were pathogenic according to the pathogenicity test; however, injury to the saffron plant was found to be a must. These fungi were pathogenic in addition to *F. oxysporum*, which is well documented as a major cause of saffron corm rot diseases in Srinagar, but in the present study, injury was a must for *F. oxysporum* as well. The percentage disease severity index for both saffron roots and corms varied for each fungal isolate.

## 1 Introduction

Corm rot disease is reported to be the major biotic stress of *Crocus sativus* (saffron), resulting in severe yield loss (Cardone et al., 2020; Kothari et al., 2021; Kumar et al., 2022a). Though various bacterial and fungal pathogens are reported to cause corm rot (Gupta et al., 2021), *Fusarium oxysporum* is reported to be the most devastating (Palmero et al., 2014; Husaini and Jiménez, 2022). After the literature survey, we discovered various reports that are the basis for making *F. oxysporum*: the report in Italy is about 28 years old (Cappelli et al., 1991), that in Japan is 68 years old (Yamamoto et al., 1954), that in India is 38 years old (Shah and Srivastava, 1984), and that in Spain is 35 years old (Garcia-Jimenez and Alfaro Garcia, 1987). Recently, several other fungal species belonging to genera *Fusarium*, *Rhizoctonia*, *Penicillium*, *Aspergillus*, *Sclerotium*, *Phoma*, *Stromatinia*, *Cochliobolus*, and *Rhizopus* have been reported to be associated with saffron diseases (Gupta et al., 2021; Mirghasempour et al., 2022b), but most of the reports suggest *F. oxysporum* as the major pathogen. In our earlier report, after isolating the fungi from the rotten corm, we also found that out of the three fungi isolated, *F. oxysporum* strain R1 was the most severe pathogen and it was different than the commonly reported *F. oxysporum* f. sp. *gladioli* (Gupta and Vakhlu, 2015). We realized that there is no recent survey on the major cause of the corm rot causing pathogens, and in some of the reports, the pathogen has been assigned the *formae speciales* without any molecular phylogeny (Gupta et al., 2011). Identification and characterization of the pathogen on the basis of the host and without molecular phylogeny is not acceptable (Bhunjun et al., 2021). Another thing that caught our attention was that the pathogens were isolated from the diseased corms and no pathogen was isolated from the diseased fields and tested for pathogenicity. Moreover, in the last 3 years, nobody has reported *F. oxysporum*, neither from rotten corms nor from the soil, as a pathogen, let alone as the most devastating pathogen (Shuwen et al., 2019; Muñoz et al., 2020; Zhang et al., 2020; Mirghasempour et al., 2022a, Mirghasempour et al., 2022b).

While unraveling the mycobiome of underground parts of saffron by metagenomics, it was found that the saffron cormosphere is dominated by Basidiomycota during the flowering stage, Zygomycota during the dormant stage in cormosphere, and Ascomycota during the vegetative stage (Ambardar et al., 2016; Bhagat et al., 2021). However, Ambardar et al. (2016) have reported that the Ascomycota is dominant in mycobiome of bulk soil. With this background, the present study aimed at unraveling the cultivable fungal diversity in traditional and non-traditional saffron cultivating fields in Jammu and Kashmir, the only region in India where saffron is cultivated at a commercial level (Ganaie and Singh, 2019; Rather et al., 2022). Furthermore, the fungal isolates were screened for pathogenicity to saffron corms and roots. In particular, the objectives of this study were as follows: (1) to collect fungal isolates from traditional and non-traditional saffron fields, (2) to evaluate the pathogenicity of the isolated fungal species against saffron, and (3) to check in real time if *F. oxysporum* is the most severe pathogen causing rot in saffron, with an aim to recommend the strategy for yield increase.

## 2 Materials and methods

### 2.1 Soil sampling, study sites, and soil analysis

**Figure 1:**
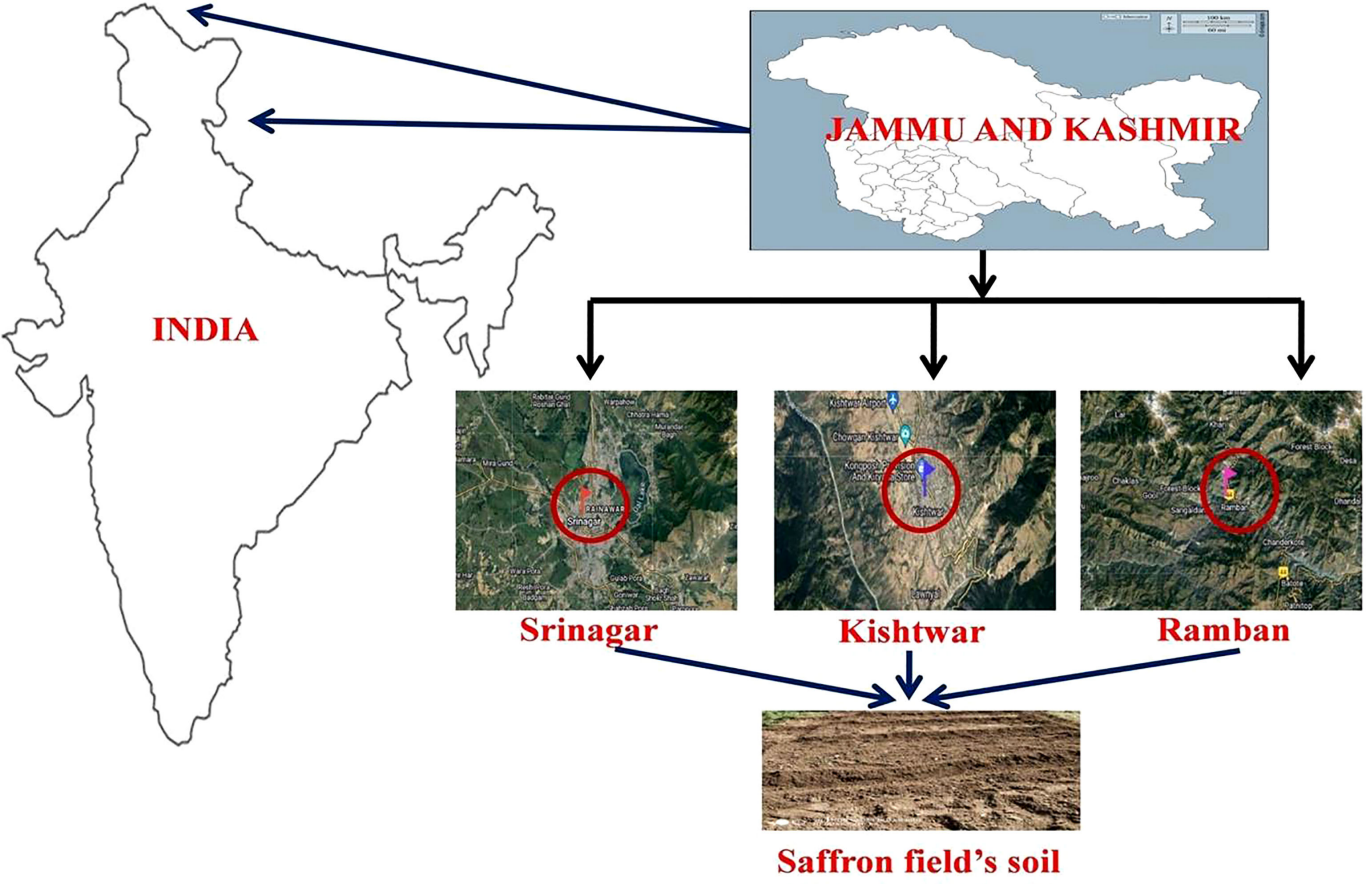
Picture representing the geographical regions (Srinagar, Kishtwar, and Ramban) of saffron fields from where soil samples were collected.

Soil samples were collected in the month of November 2018 from the three sampling sites—traditional saffron fields: Srinagar (Pampore)-34.02°N 74.93°E and Kishtwar-33.32°N 75.77°E and a non-traditional saffron field: Ramban-33.25°N 75.25°E (**Figure 1**). For each sampling site, soil samples were collected randomly from different spots, homogenized, and appropriately mixed to form one sample. The soil samples of three locations were sieved to remove the non-soil components such as plant and animal leftovers and pebbles. Each soil sample was then divided into three major parts: (i) the first part of the soil was preserved at −20°C, (ii) another part was used for the determination of the soil’s physio-chemical properties, and (iii) the last fraction of each soil sample was preserved at 4°C for the culture-dependent fungal communities’ analysis. The soil sample of three geographic locations was sent to Yara Fertilisers India Pvt. Ltd. Analysis for the analysis of standard physio-chemical parameters of soil. The “Standard” soil analysis included analysis and interpretation of the parameters–pH, electrical conductivity (EC, mmhos/cm), organic carbon (OC, %), nitrogen (N, kg/ha), phosphorus (P, kg/ha), potassium (K, kg/ha), sulfur (S, ppm), zinc (Zn, ppm), and boron (B, ppm). The results have been generated by Yara’s Megalab™ software.

### 2.2 Isolation of fungi from the saffron fields

The isolation of fungi was done from all the three location soils, so as to identify the inhabitant fungus up to species/strain level. The serial dilution plating method was carried out for the dilution of soil samples as mentioned by Waksman (1992), with the purpose of avoiding overcrowding of fungal colonies in the soil in each dilution (Waksman, 1992). Two replicates were used for each dilution of each 1 g of soil sample up to 10^−12^ dilution. One hundred microliters (0.1 ml) of each dilution was plated on four different media: (1) Potato Dextrose Agar (PDA), (2) Czapek Dox Agar (CDA), (3) Sabouraud Dextrose Agar (SDA), and (4) Peptone pentachloronitrobenzene agar media (PPA). Chloramphenicol (25 mg/ml) was added to all media before pouring into Petri plates for preventing bacterial growth. The Petri plates were then incubated at 28 ± 2°C for 4–7 days. Each colony type thus obtained was transferred to a new PDA plate for macroscopic, morphological, and molecular identification. The maintenance of pure cultures was done by sub-culturing by incubating the plates at 28 ± 2°C.

### 2.3 DNA extraction and soil fungal isolate identification

The genomic DNA was extracted according to the method of Saghai-Maroof et al. (1984). The fresh mycelium of fungal cultures grown on PDA at 25°C for 7 days was used for DNA isolation. For the preliminary identification of the fungal colonies, the primer pair ITS1/ITS4—ITS1 (5′-TCCGTAGGTGAACCTGCGG-3′) and ITS4 (5′-TCCTCCGCTTATTGATATGC-3′)—was used for amplification and sequencing of the ITS region (Gupta and Vakhlu, 2015). The 10-μl PCR reaction contained the following: 5.3 μl of nuclease-free water (HIMEDIA, ML064), 1 μl of Kappa Taq buffer (10X), 0.1 μl of MgCl_2_ (25 mM), 0.4 μl of dNTPs (10 mM) (Promega U1518), 0.2 μl of Kappa Taq (5 U/μl) (KAPA Taq PCR KIT, KK1015), 1 μl of each primer (10 μM), and 1 μl (1,000 ng/μl) of each fungal DNA sample. The amplification was performed as follows: denaturation at 95°C for 5 min, followed by 35 cycles of 95°C for 30 s, annealing at 55°C for 1 min, and extension at 72°C for 1 min, with a final extension at 72°C for 7 min. The PCR amplicons were verified on 1% agarose gel electrophoresis by loading 4 µl of the PCR product and 2 µl of a reference ladder (0.5 μg/μl). The PCR products were outsourced for Sanger sequencing to Biologia Research Pvt. Ltd. (Haryana, India) and sequences were analyzed by BLAST (Basic Local Alignment Search Tool) (https://blast.ncbi.nlm.nih.gov/Blast.cgi).

### 2.4 Diversity analysis for fungal isolates

The alpha diversity was calculated as species richness and was measured for all the three sampling locations. The species richness was defined as the total number of species isolated from the particular soil location. The formula H′ = − Σ(Pi × InPi), where Pi denotes the proportional abundance of fungal isolates in a specific location, was used to calculate Shannon’s H′ diversity index (Ricotta, 2002). The relative abundance was estimated as a percentage of a fungal isolate belonging to a specific species/group as compared to the total number of isolates (Jan et al., 2022). All the diversity analyses were performed using PAST 3.04 software (Hammer et al., 2001).

### 2.5 Pathogenicity tests

#### 2.5.1 Preparation of saffron corm for the pathogenicity test

The pathogenicity test of 45 fungal isolates on the saffron plant was conducted in pots. *F. oxysporum* R1 (Fox R1) earlier reported as a pathogenic strain to saffron corms was used as a reference point for pathogenicity (Gupta and Vakhlu, 2015). For negative control, plant inoculation with autoclaved distilled water was used. Healthy corms were disinfected by dipping them in 5% of sodium hypochlorite solution for 15 min. This was followed by three washes with autoclaved distilled water. The corms were planted in sterile plastic pots (17 cm diameter) containing two-thirds autoclaved sand and soil at a ratio of 1:1 (Palmero et al., 2014). The test was conducted in five different combinations with three replicates of each, and the combinations are tabulated in **Table 1** for easy comprehension. For the corm injury, the apical end of corm was injured with autoclaved pipette tip (HIMEDIA, Pipette tips, Neutral color, 0.5–10 μl—CG308-1x1000NO) causing a 10-mm-deep and 2-mm-wide injury (Bhagat et al., 2022). For root injury, the tips of roots were cut 1 cm from the terminal end with sterile scissors.

**Table 1:** Injury parameters selected for the pathogenicity test of the fungal isolates.

Test parameters	Injury
Parameter 1	No injury was given
Parameter 2	Corms were injured
Parameter 3	Roots were injured
Parameter 4	Both the corms and roots were injured
Parameter 5	Surface scarping of the corms was done

#### 2.5.2 Preparation of fungal inoculums for the pathogenicity test

The fungi inoculums were grown in potato dextrose broth at 27°C with constant shaking at 150 rpm in the incubator (ORBITEK). The inoculums were prepared by filtering 7-day-old fungal culture through double-layer cheesecloth. This was done to remove the mycelium from the inoculums to be used. For each isolate, the conidia suspension was maintained at 10^7^ conidia/ml. One milliliter of each conidia suspension was mixed with the sand–soil combination in the pots and left for 1 month with controlled light and temperature conditions (12/12 h light/dark; 25/21°C) (Gupta and Vakhlu, 2015; Wani et al., 2016). Plants were watered regularly during this period to keep the soil–sand mixture moist. The presence and absence of rot symptoms on corm and root were evaluated after 10, 20, and 30 days of inoculation, respectively.

The pathogenicity and capacity of each isolate to cause rot symptoms on roots and corms were demonstrated according to Avan et al. (2021). The roots were assessed according to the rot symptoms defined on a 0–3 scale: 0 = no disease, 1 = one-third of the roots are necrotic, 2 = two-thirds of the roots are necrotic, 3 = all of the roots are necrotic. The corm rot symptoms were estimated by cutting the corm in half along their longer axes and two perpendicular diameters of internal lesions were determined. The mean of two diameters represented the lesion size of each corm (Azil et al., 2021). The rating index used for the evaluation of corm rotting was recorded according to the following: 0 = no symptoms of corm rot, 1 = rotting near point of injury, 2 = rotting inside the corm, 3 = rotting both inside and outside the corm, and 4 = whole corm rot (Wani et al., 2016). The disease severity index (DSI) values were calculated for each fungal isolate using the following formula as per Avan et al. (2021).


$$DSI=\frac{\sum \left(NS\times SV\right)}{HSV\times TS}$$

DSI—disease severity index     NS—number of seedlings at each scale

SV—scale value           HSV—the highest scale value

TS—total seedling evaluated

The percent disease severities were estimated by multiplying the value of disease severity index with 100. The pathogenicity profiling of fungal isolates was grouped into four categories: NP—non-pathogenic (below 40% disease severity); LP—low pathogenic (41%–60% disease severity); MP—moderately pathogenic (61%–80% disease severity); and HP—highly pathogenic (81%–100% disease severity). For the final evaluation of symptoms, 10, 20, and 30 days post-inoculation (dpi), five plants were chosen for five injury treatments in triplicates for each isolate (5 × 3 = 15 plants in total), and the experiment was conducted twice. Tissue sections with symptoms were used for the re-isolation of pathogens, and to confirm Koch’s postulates, recovered isolates were compared to primarily inoculated pathogens.

### 2.6 Statistical analysis

**Figure 2:**
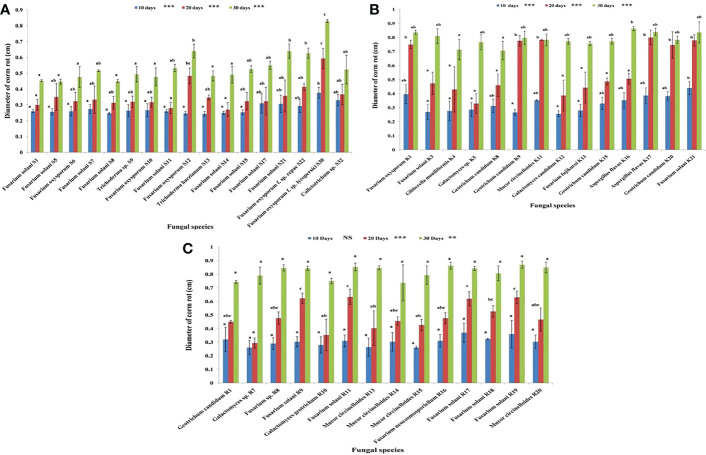
**(A)** Diameter of corm rot (in cm) caused by fungal species isolated from Srinagar soil at 10, 20, and 30 days post-inoculation. **(B)** Diameter of corm rot (in cm) caused by fungal species isolated from Kishtwar soil at 10, 20, and 30 days post-inoculation. **(C)** Diameter of corm rot (in cm) caused by fungal species isolated from Ramban soil at 10, 20, and 30 days post- inoculation. Mean diameters in the same day bar followed by lowercase letter(s) are significantly different at *p* ≤ 0.05, according to Tukey’s test. *, significance at *p*< 0.05; **, significance at *p*< 0.01; ***, significance at *p*< 0.001; NS, no significant difference.

For corm rot pathogenicity results, the lesion sizes of every individual corm were analyzed statistically. The recorded data were subjected to one-way analysis of variance (ANOVA) using IBM SPSS statistics version 26. The significance of mean differences in lesion sizes was observed using Tukey’s test at 5% level of threshold significance. The data presented in **Figures 2A–C** depict mean values ± standard deviation (SD) for diameter of corm rot. In **Figure 2**, the mean diameter in the same day bar followed by lowercase letter(s) represents significant difference at *p* ≤ 0.05, according to Tukey’s test. The single asterisk (*) represents significant difference at *p*< 0.05, the double asterisk (**) represents significant difference at *p*< 0.01, and the triple asterisk (***) represents significant difference at *p*< 0.001; NS represents no significant difference.

## 3 Results

### 3.1 Soil analysis and diversity of fungal isolates

The location and various physio-chemical properties of the soil of the sampling sites are presented in **Table 2**. The soil of all the selected locations has an alkaline pH ranging from 7.6 to 8.5. The Srinagar (Pampore) soil has the highest pH of 8.5; however, the EC, OC, N, P, K, S, Zn, and B were comparatively lower than the other two locations, i.e., Kishtwar and Ramban. The EC, OC, S, Zn, and B contents were highest in Ramban soil, whereas the other soil contents such as N, P, and K were highest in the Kishtwar soil. A total of 45 fungal isolates were isolated from saffron fields across three different study locations (**Figure 1**). Based on ITS rDNA gene sequences followed by BLASTn analysis, these isolates were resolved into 45 operational taxonomic units (OTUs) distributed across seven genera, i.e., *Fusarium*, *Geotrichum*, *Mucor*, *Aspergillus*, *Galactomyces*, *Trichoderma*, and *Colletotrichum*. Majority of these fungal isolates represented >99% similarity with the reference strains. The ITS sequences have been deposited in GenBank, and accession numbers have been tabulated in **Table 3**. The phylogenetic relationship of 45 OTUs corresponded to a broad taxonomic range. The phylogenetic topology revealed that Ascomycota and Mucoromycota were the two phyla into which the entire representative isolates could be grouped (**Supplementary Figure 1**). Ascomycota was predominant, accounting for 88.88% of total OTUs, followed by Mucoromycota at 11.11%. *Fusarium*, a member of the Ascomycota phylum, was the most prevalent genus among all the identified genera distributed across these two phyla, accounting for 57.77% of all OTUs. *Geotrichum*, a genera of Ascomycota phylum, and *Mucor* of the phylum Mucoromycota accounted for approximately 17.77% and 11.11% of all OTUs, respectively. Both *Aspergillus* and *Trichoderma* belonging to the Ascomycota phylum were about 4.44% abundant. Out of all the seven genera, *Galactomyces* and *Colletotrichum* were the least abundant (2.22%) (**Supplementary Figure 2**).

**Table 2:** Location of sampling sites and their soil properties.

Sampling site	Location coordinates	Soil properties								
		pH	Electrical conductivity (EC, mmhos/cm)	Organic carbon (OC, %)	Nitrogen (N, kg/ha)	Phosphorus (P, kg/ha)	Potassium (K, kg/ha)	Sulfur (S, ppm)	Zinc (Zn, ppm)	Boron (B, ppm)
**Srinagar (Pampore)**	**34.02°N 74.93°E**	8.5	0.17	0.38	70.37	24.6	215.9	9	0.46	0.09
**Kishtwar**	**33.32°N 75.77°E**	7.9	0.29	1.47	192.37	105.3	789.3	12	3.20	0.63
**Ramban**	**33.25°N 75.25°E**	7.6	0.67	1.80	157.15	31.4	288.3	21	3.68	0.91

**Table 3:** Description of the OTUs representing the fungal species isolated from the three geographic locations of saffron fields (Srinagar, Kishtwar, and Ramban).

S. no.	Isolate name	Closest match in GenBank	Assigned accession no.	% Similarity
**1.** **2.** **3.** **4.** **5.** **6.** **7.** **8.** **9.** **10.** **11.** **12.** **13.** **14.** **15.** **16.** **17.**	**Srinagar** ** S1** ** S5** ** S6** ** S7** ** S8** ** S9** ** S10** ** S11** ** S12** ** S13** ** S14** ** S15** ** S17** ** S21** ** S22** ** S30** ** S32**	***Fusarium solani*** ***Fusarium solani*** ***Fusarium oxysporum*** ***Fusarium solani*** ***Fusarium solani*** ***Trichoderma* sp.** ***Fusarium oxysporum*** ***Fusarium solani*** ***Fusarium oxysporum*** ***Trichoderma harzianum*** ***Fusarium solani*** ***Fusarium solani*** ***Fusarium solani*** ***Fusarium solani*** ***Fusarium oxysporum* f. sp. *cepae*** ***Fusarium oxysporum* f. sp. *lycopersici*** ***Colletotrichum* sp.**	**OP349605** **OP349599** **OP349597** **OP329709** **OP349596** **OP348885** **OP341431** **OP348876** **OP348869** **OP348900** **OP341450** **OP341616** **OP341621** **OP348901** **OP347088** **OP347081** **OP346968**	**100%** **100%** **100%** **96.60%** **100%** **100%** **93.26%** **100%** **100%** **100%** **96.54%** **96.02%** **90.68%** **100%** **100%** **100%** **100%**
**1.** **2.** **3.** **4.** **5.** **6.** **7.** **8.** **9.** **10.** **11.** **12.** **13.** **14.**	**Kishtwar** ** K1** ** K3** ** K4** ** K5** ** K8** ** K9** ** K11** ** K12** ** K13** ** K15** ** K16** ** K17** ** K20** ** K21**	***Fusarium oxysporum*** ***Fusarium solani*** ***Gibberella moniliformis*** ***Galactomyces* sp.** ***Geotrichum candidum*** ***Geotrichum candidum*** ***Mucor circinelloides*** ***Galactomyces candidum*** ***Fusarium fujikuroi*** ***Geotrichum candidum*** ***Aspergillus flavus*** ***Aspergillus flavus*** ***Geotrichum candidum*** ***Fusarium solani***	**OP349665** **OP349663** **OP349635** **OP349662** **OP318066** **OP349661** **OP317630** **OP349653** **OP349639** **OP349636** **OP349633** **OP349634** **OP318067** **OP320349**	**100%** **100%** **100%** **100%** **92.86%** **100%** **91.33%** **100%** **100%** **100%** **100%** **100%** **88.92%** **95.07%**
**1.** **2.** **3.** **4.** **5.** **6.** **7.** **8.** **9.** **10.** **11.** **12.** **13.** **14.**	**Ramban** ** R1** ** R7** ** R8** ** R9** ** R10** ** R11** ** R13** ** R14** ** R15** ** R16** ** R17** ** R18** ** R19** ** R20**	***Geotrichum candidum*** ***Galactomyces* sp.** ***Fusarium* sp.** ***Fusarium solani*** ***Galactomyces geotrichum*** ***Fusarium solani*** ***Mucor circinelloides*** ***Mucor circinelloides*** ***Mucor circinelloides*** ***Fusarium neocosmosporiellum*** ***Fusarium solani*** ***Fusarium solani*** ***Fusarium solani*** ***Mucor circinelloides***	**OP349631** **OP324630** **OP349632** **OP349666** **OP324645** **OP349623** **OP349624** **OP326282** **OP326237** **OP349618** **OP349617** **OP349613** **OP326281** **OP349607**	**100%** **99.11%** **100%** **99.51%** **96.88%** **100%** **100%** **97.71%** **89.45%** **100%** **100%** **100%** **91.35%** **100%**

### 3.2 Diversity and distribution of fungal isolates across different locations

**Figure 3:**
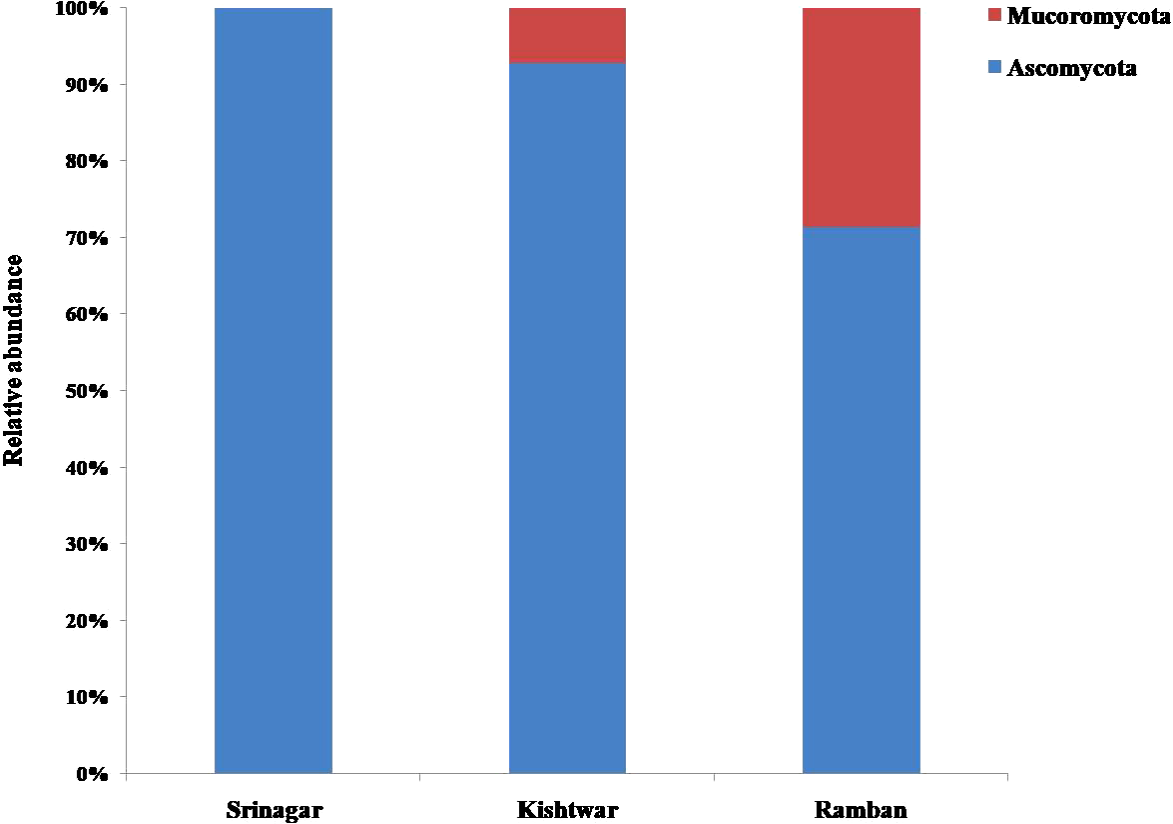
Relative abundance of individual operational taxonomic units (OTUs) at the phylum level across the three locations (Srinagar, Kishtwar, and Ramban).

**Figure 4:**
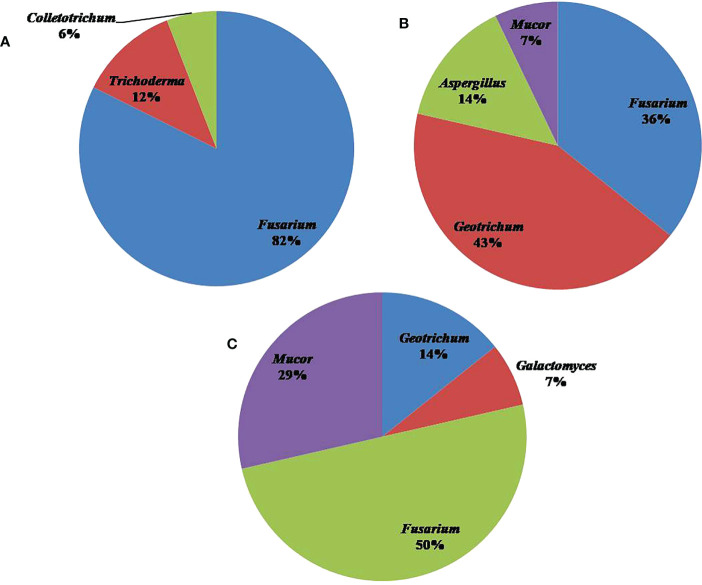
Pie chart representing the distribution and diversity of fungal genera in **(A)** Srinagar, **(B)** Kishtwar, and **(C)** Ramban.

Among different locations, Pampore in Srinagar harbored the highest number of fungal isolates, i.e., 17, followed by Kishtwar and Ramban with 14 isolates each. The different location sites yielded variable results vis-a-vis the genera of fungal isolates. OTUs belonging to Ascomycota constituted the most abundant fungal isolates in all the three locations followed by Mucoromycota, but it was absent in Srinagar (**Figure 3**). The four genera present in Kishtwar soil were *Fusarium*, *Geotrichum*, *Mucor*, and *Aspergillus*. In Ramban soil, *Fusarium,Geotrichum*, *Mucor*, and *Galactomyces* were the four genera observed. Notably, only three genera, *Fusarium*, *Trichoderma*, and *Colletotrichum*, were isolated from Srinagar soil (**Figures 4A–C**). The four genera *Geotrichum*, *Aspergillus*, *Mucor*, and *Galactomyces* were absent in Srinagar but were isolated from the other two locations. The genera *Trichoderma* and *Colletotrichum* were exclusively isolated from Srinagar soil. However, *Aspergillus* was found only in Kishtwar. The genus *Fusarium* was present in all the three locations. Srinagar reported highest OTUs of *Fusarium* genera, i.e., 82.35% out of all the OTUs observed in Srinagar, followed by *Trichoderma* and *Colletotrichum*, accounting for 11.76% and 5.88% OTUs, respectively. *Geotrichum* and *Fusarium* were found to be abundant, i.e., 42.85% and 35.71% OTUs out of all the fungal isolates isolated from Kishtwar soil. However, *Aspergillus* and *Mucor* were lower in percentage, i.e., 14.28% and 7.14%, respectively, in Kishtwar. In Ramban, *Fusarium* and *Mucor* were the highly representative genera, with 50.00% and 28.57% OTUs present, respectively, of all the OTUs obtained in the Ramban region. The other two genera, namely, *Geotrichum* and *Galactomyces*, were 14.28% and 7.14% abundant, respectively (**Figures 4A–C**).

**Figure 5:**
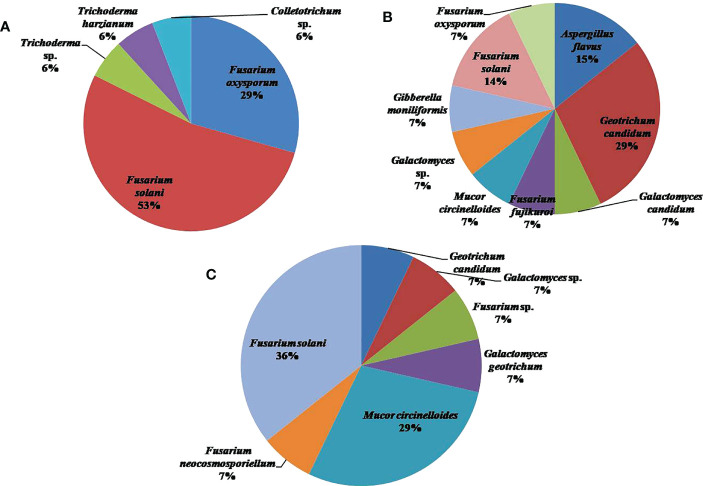
Pie chart representing the distribution and diversity of fungal species in **(A)** Srinagar, **(B)** Kishtwar, and **(C)** Ramban.

In Kishtwar, the 14 species isolated were *Geotrichum candidum* (28.57%; *n* = 4), *Fusarium solani* (14.28%; *n* = 2), *Aspergillus flavus* (14.28%; *n* = 2), *Galactomyces candidum* (7.14%; *n* = 1), *Fusarium fujikuroi* (7.14%; *n* = 1), *Mucor circinelloides* (7.14%; *n* = 1), *Galactomyces* sp. (7.14%; *n* = 1), *Gibberella moniliformis* (7.14%; *n* = 1), and *F. oxysporum* (7.14%; *n* = 1). The species that were found in the Ramban soil were *F. solani* (35.71%; *n* = 5), *M. circinelloides* (28.57%; *n* = 4), *G. candidum* (7.14%; *n* = 1), *Galactomyces* sp. (7.14%; *n* = 1), *Fusarium* sp. (7.14%; *n* = 1), *Galactomyces geotrichum* (7.14%; *n* = 1), and *Fusarium neocosmosporiellum* (7.14%; *n* = 1). In the Srinagar soil, the fungal isolates corresponded to the following species: *F. solani* (52.94%; *n* = 9), *F. oxysporum* (29.41%; *n* = 5), *Trichoderma* sp. (5.88%; *n* = 1), *Trichoderma harzianum* (5.88%; *n* = 1), and *Colletotrichum* sp. (5.88%; *n* = 1). The graphic representation of this diversity is given in **Figures 5A–C**.

**Figure 6:**
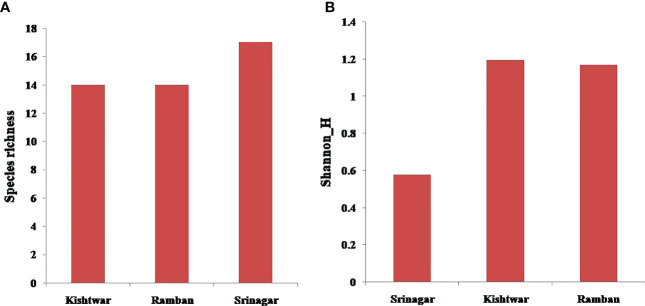
**(A)** Species richness across the three locations (Srinagar, Kishtwar and Ramban). **(B)** Shannon’s (H′) index across the three locations (Srinagar, Kishtwar, and Ramban).

It was observed that Srinagar soil recorded the highest species richness with 17 OTUs, followed by Kishtwar and Ramban with 14 OTUs each (**Figure 6A**). The Shannon (H′) index was highest for the Kishtwar region (H′= 1.197), followed by Ramban (H′= 1.171). The Srinagar location had the lowest H′ index of 0.5783 (**Figure 6B**).

### 3.3 Pathogenicity test of fungal isolates

All the fungal isolates were found to be pathogenic on saffron corms as well as on roots, but only when they were artificially injured. Non-injured and injured corm/root in the absence of fungal inoculums remained healthy and did not develop any visible symptoms during the experiment. However, the pathogenicity results of all the 45 fungal isolates had variability in the range of percent disease severity index.

#### 3.3.1 Corms rot profiling

**Figure 7:**
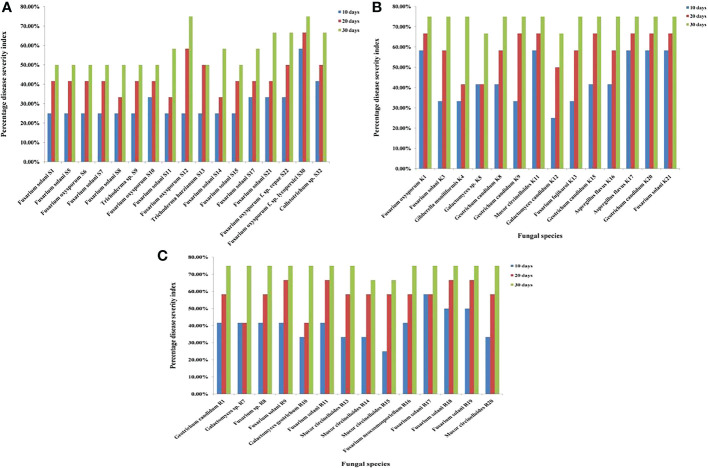
**(A)** Pathogenicity test of fungal species isolated from Srinagar soil on saffron corms at 10, 20, and 30 days post-inoculation (dpi). **(B)** Pathogenicity test of fungal species isolated from Kishtwar soil on saffron corms at 10, 20 and 30 dpi. **(C)** Pathogenicity test of fungal species isolated from Ramban soil on saffron corms at 10, 20, and 30 dpi.

Pathogenicity profiling was grouped into four categories and was estimated on both corms and roots in correspondence to the point of fungal inoculation. The corm rot symptoms were observed for 1 month to follow the progress of disease symptoms after inoculation with each isolate. At 10 days post-inoculation (dpi), almost all the fungal isolates looked either non-pathogenic or low pathogenic as symptoms were invisible or mildly visible on the saffron corm. However, from 20 dpi to 30 dpi, disease severity index increased from the category of low pathogenic to moderately pathogenic. Of the Ramban soil fungal isolates, five fungal isolates were non-pathogenic, including four *M. circinelloides* strains and one *G. geotrichum* strain at 10 dpi. Conversely, nine other fungal strains such as *G. candidum*, *Galactomyces* sp., *F. solani* (5), *Fusarium* sp., and *F. neocosmosporiellum* were low pathogenic at 10 dpi. All these fungal isolates were moderately pathogenic at 30 dpi (up to 75% disease severity index). Similarly, all the fungal strains isolated from the Kishtwar soil were also moderately pathogenic, showing maximum of 75% disease severity index at 30 dpi to the saffron corm. In contrast to Ramban and Kishtwar, most of the fungal strains isolated from the Srinagar soil were low pathogenic at 30 dpi. *F. oxysporum* (3), *F. solani* (1), and *Colletotrichum* sp. (1) were the five strains among the 17 reported in Srinagar that were moderately pathogenic to the saffron corm at 30 dpi (**Table 4**; **Figures 7A–C**). *F. oxysporum* R1, which was considered as positive control, was non-pathogenic at 10 dpi (25% DSI); however, percentage disease severity index was increased to 50% at 20 dpi and 66.66% at 30 dpi, respectively. The three out of six *F. oxysporum* that were isolated in the present study were more pathogenic than *F. oxysporum* R1, as they exhibited 75% DSI at 30 dpi. These three *F. oxysporum* species were named *F. oxysporum* S12*, F. oxysporum* f. sp. *lycopersici* S30, and *F. oxysporum* K1.

**Table 4:** Corm rot pathogenicity profiling of fungal species isolated from Srinagar, Kishtwar, and Ramban location.

Fungal isolate name**		Disease severity index (%) (10 days post-inoculation)	Virulence* (10 days post-inoculation)	Disease severity index (%) (20 days post-inoculation)	Virulence* (20 days post-inoculation)	Disease severity index (%) (30 days post-inoculation)	Virulence* (30 days post-inoculation)
Srinagar							
**S1** **S5** **S6** **S7** **S8** **S9** **S10** **S11** **S12** **S13** **S14** **S15** **S17** **S21** **S22** **S30** **S32**	***Fusarium solani*** ***Fusarium solani*** ***Fusarium oxysporum*** ***Fusarium solani*** ***Fusarium solani*** ***Trichoderma* sp.** ***Fusarium oxysporum*** ***Fusarium solani*** ***Fusarium oxysporum*** ***Trichoderma harzianum*** ***Fusarium solani*** ***Fusarium solani*** ***Fusarium solani*** ***Fusarium solani*** ***Fusarium oxysporum* f. sp. *cepae*** ***Fusarium oxysporum* f. sp. *lycopersici*** ***Colletotrichum* sp.**	25.00% 25.00% 25.00% 25.00% 25.00% 25.00% 33.33% 25.00% 25.00% 25.00% 25.00% 25.00% 33.33% 33.33% 33.33% 58.33% 41.66%	**NP** **NP** **NP** **NP** **NP** **NP** **NP** **NP** **NP** **NP** **NP** **NP** **NP** **NP** **NP** **LP** **LP**	41.66% 41.66% 41.66% 41.66% 33.33% 41.66% 41.66% 33.33% 58.33% 50.00% 33.33% 41.66% 41.66% 41.66% 50.00% 66.66% 50.00%	**LP** **LP** **LP** **LP** **NP** **LP** **LP** **NP** **LP** **LP** **NP** **LP** **LP** **LP** **LP** **MP** **LP**	50.00% 50.00% 50.00% 50.00% 50.00% 50.00% 50.00% 58.33% 75.00% 50.00% 58.33% 50.00% 58.33% 66.66% 66.66% 75.00% 66.66%	**LP** **LP** **LP** **LP** **LP** **LP** **LP** **LP** **MP** **LP** **LP** **LP** **LP** **MP** **MP** **MP** **MP**
Kishtwar							
**K1** **K3** **K4** **K5** **K8** **K9** **K11** **K12** **K13** **K15** **K16** **K17** **K20** **K21**	***Fusarium oxysporum*** ***Fusarium solani*** ***Gibberella moniliformis*** ***Galactomyces* sp.** ***Geotrichum candidum*** ***Geotrichum candidum*** ***Mucor circinelloides*** ***Galactomyces candidum*** ***Fusarium fujikuroi*** ***Geotrichum candidum*** ***Aspergillus flavus*** ***Aspergillus flavus*** ***Geotrichum candidum*** ***Fusarium solani***	58.33% 33.33% 33.33% 41.66% 41.66% 33.33% 58.33% 25.00% 33.33% 41.66% 41.66% 58.33% 58.33% 58.33%	**LP** **NP** **NP** **LP** **LP** **NP** **LP** **NP** **NP** **LP** **LP** **LP** **LP** **LP**	66.66% 58.33% 41.66% 41.66% 58.33% 66.66% 66.66% 50.00% 58.33% 66.66% 58.33% 66.66% 66.66% 66.66%	**MP** **LP** **LP** **LP** **LP** **MP** **MP** **LP** **LP** **MP** **LP** **MP** **MP** **MP**	75% 75% 75% 66.66% 75% 75% 75% 66.66% 75% 75% 75% 75% 75% 75%	**MP** **MP** **MP** **MP** **MP** **MP** **MP** **MP** **MP** **MP** **MP** **MP** **MP** **MP**
Ramban							
**R1** **R7** **R8** **R9** **R10** **R11** **R13** **R14** **R15** **R16** **R17** **R18** **R19** **R20**	***Geotrichum candidum*** ***Galactomyces* sp.** ***Fusarium* sp.** ***Fusarium solani*** ***Galactomyces geotrichum*** ***Fusarium solani*** ***Mucor circinelloides*** ***Mucor circinelloides*** ***Mucor circinelloides*** ***Fusarium neocosmosporiellum*** ***Fusarium solani*** ***Fusarium solani*** ***Fusarium solani*** ***Mucor circinelloides***	41.66% 41.66% 41.66% 41.66% 33.33% 41.66% 33.33% 33.33% 25.00% 41.66% 58.33% 50.00% 50.00% 33.33%	**LP** **LP** **LP** **LP** **NP** **LP** **NP** **NP** **NP** **LP** **LP** **LP** **LP** **NP**	58.33% 41.66% 58.33% 66.66% 41.66% 66.66% 58.33% 58.33% 58.33% 58.33% 58.33% 66.66% 66.66% 58.33%	**LP** **LP** **LP** **MP** **LP** **MP** **LP** **LP** **LP** **LP** **LP** **MP** **MP** **LP**	75% 75% 75% 75% 75% 75% 75% 66.66% 66.66% 75% 75% 75% 75% 75%	**MP** **MP** **MP** **MP** **MP** **MP** **MP** **MP** **MP** **MP** **MP** **MP** **MP** **MP**
**Fox R1** **Accession No. KF663598** **(Positive Control)**	***Fusarium oxysporum***	25%	**NP**	50%	**LP**	66.66%	**MP**

* NP, non-pathogenic (below 40% disease severity); LP, low pathogenic (41%–60% disease severity); MP, moderately pathogenic (61%–80% disease severity); and HP, highly pathogenic (81%–100% disease severity). **Fungal species name are presented in the same order as fungal isolate name.

The mean of lesion sizes formed by fungal species were uneven, ranging from the 0.24 cm by *T. harzianum* S13 to 0.44 cm by *F. solani* K21 at 10 dpi. Moreover, at 20 dpi, the lesion size further increased from 0.27 cm by *F. solani* S14 to 0.80 cm by *A. flavus* K17. At 30 dpi, the lesion size was observed in the range of 0.44 cm by *F. solani* S5 to 0.87 cm by *F. solani* R19. It was observed that the diameter of the lesion was in the range of 0.26–0.37 cm at 10 dpi, 0.29–0.63 cm at 20 dpi, and 0.73–0.87 cm at 30 dpi for the fungal isolates obtained from Ramban soil. However, the variable range of 0.25–0.44 cm at 10 dpi, 0.33–0.80 cm at 20 dpi, and 0.70–0.86 cm at 30 dpi was observed in case of Kishtwar. In contrast to Ramban and Kishtwar isolates, Srinagar isolates had a lesion size estimated at 0.24–0.37 cm at 10 dpi, 0.27–0.59 cm at 20 dpi, and 0.44–0.83 cm at 30 dpi (**Figures 2A–C**).

#### 3.3.2 Root rot profiling

**Figure 8:**
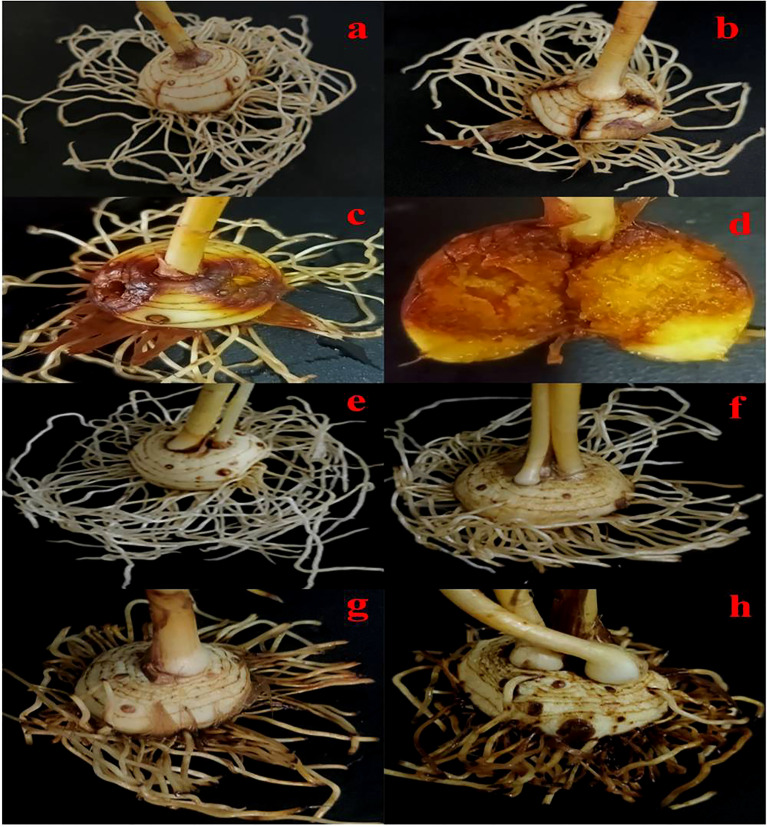
Symptoms of rot on saffron corms and roots caused by fungal isolates. **(A)** Poorly developed symptoms on corms, **(B)** moderate symptoms of corm rot, **(C,D)** severe corm rot, **(E)** no symptoms of saffron root rot, **(F)** poorly developed symptoms on roots, **(G)** moderate symptoms of root rot, and **(H)** severe root rot.

**Figure 9:**
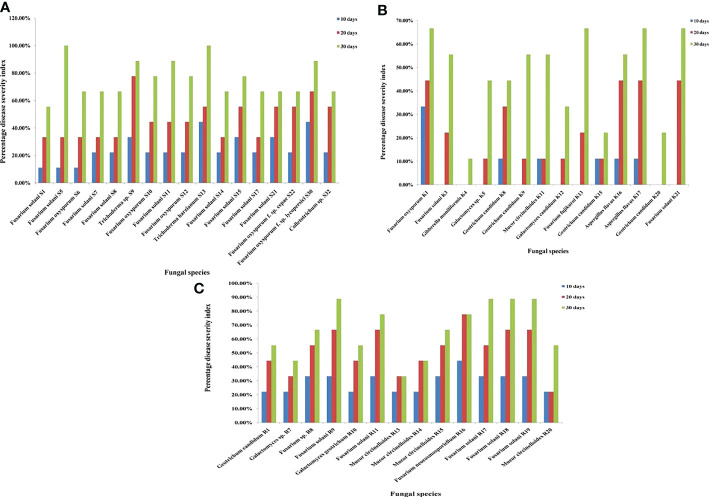
**(A)** Pathogenicity test of fungal species isolated from Srinagar soil on saffron roots at 10, 20, and 30 days post-inoculation (dpi). **(B)** Pathogenicity test of fungal species isolated from Kishtwar soil on saffron roots at 10, 20, and 30 dpi. **(C)** Pathogenicity test of fungal species isolated from Ramban soil on saffron roots at 10, 20, and 30 dpi.

As in the case of corms, in roots as well, all the inoculated fungal isolates exhibited slight browning of roots as the initial symptoms. Eventually, the progression of infection resulted in browning, black necrotic lesions, and wilting of the roots (**Figure 8**). All the isolates were in non-pathogenic category at 10 dpi with the exception of *F. neocosmosporiellum* R16 with 44.44% disease severity, *T. harzianum* S13 with 44.44%, and *F. oxysporum* f. sp. *lycopersici* S30 with 44.44%. At 20 dpi, fungal isolates could be divided into a mixed proportion of non-pathogenic, low pathogenic, and moderately pathogenic categories. Highly pathogenic isolates were reported only in Ramban and Srinagar soil at 30 dpi. The four *F. solani* strains, namely, R9, R17, R18, and R19, had 88.88% disease severity index at 30 dpi; hence, they were highly pathogenic strains identified in Ramban soil. However, highly pathogenic strains obtained from Srinagar at 30 dpi were *T. harzianum* S13 (100%), *F. solani* S5 (100%), *Trichoderma* sp. S9 (88.88%), *F. solani* S11 (88.88%), and *F. oxysporum* f. sp. *lycopersici* S30 (88.88%) (**Table 5**; **Figures 9A–C**). The already reported *F. oxysporum* R1 taken as a positive control was as pathogenic as *F. oxysporum* f. sp. *lycopersici* S30 that was isolated in the present study. *F. oxysporum* R1 resulted in 88.88% disease severity at 30 dpi, similar to *F. oxysporum* f. sp. *lycopersici* S30. However, *F. oxysporum* R1 was more pathogenic to saffron roots than other *F. oxysporum* species isolated in the study but not as pathogenic as *T. harzianum* S13 that affected roots more severely, i.e., 100% DSI at 30 dpi. The pathogenicity of all the isolates was confirmed by Koch’s postulates after their re-isolation from the symptomatic tissues, and the identities of the re-isolate species were confirmed using microscopic, macroscopic, and molecular analysis fulfilling Koch’s postulates.

**Table 5:** Root rot pathogenicity profiling of fungal species isolated from Srinagar, Kishtwar and Ramban location.

Fungal isolates name**		Disease severity index (%)(10 days Post inoculation)	Virulence*(10 days Post inoculation)	Disease severity index (%)(20 days post inoculation)	Virulence*(20 days post inoculation)	Disease severity index (%)(30 days post inoculation)	Virulence*(30 days post inoculation)
**Srinagar**							
**S1**	***Fusarium solani***	11.11%	**NP**	33.33%	**NP**	55.55%	**LP**
**S5**	***Fusarium solani***	11.11%	**NP**	33.33%	**NP**	100%	**HP**
**S6**	***Fusarium oxysporum***	11.11%	**NP**	33.33%	**NP**	66.66%	**MP**
**S7**	***Fusarium solani***	22.22%	**NP**	33.33%	**NP**	66.66%	**MP**
**S8**	***Fusarium solani***	22.22%	**NP**	33.33%	**NP**	66.66%	**MP**
**S9**	***Trichoderma* sp.**	33.33%	**NP**	77.77%	**MP**	88.88%	**HP**
**S10**	***Fusarium oxysporum***	22.22%	**NP**	44.44%	**LP**	77.77%	**MP**
**S11**	***Fusarium solani***	22.22%	**NP**	44.44%	**LP**	88.88%	**HP**
**S12**	***Fusarium oxysporum***	22.22%	**NP**	44.44%	**LP**	77.77%	**MP**
**S13**	***Trichoderma harzianum***	44.44%	**LP**	55.55%	**LP**	100%	**HP**
**S14**	***Fusarium solani***	22.22%	**NP**	33.33%	**NP**	66.66%	**MP**
**S15**	***Fusarium solani***	33.33%	**NP**	55.55%	**LP**	77.77%	**MP**
**S17**	***Fusarium solani***	22.22%	**NP**	33.33%	**NP**	66.66%	**MP**
**S21**	***Fusarium solani***	33.33%	**NP**	55.55%	**LP**	66.66%	**MP**
**S22**	***Fusarium oxysporum* f. sp. *cepae***	22.22%	**NP**	55.55%	**LP**	66.66%	**MP**
**S30**	***Fusarium oxysporum* f. sp. *lycopersici***	44.44%	**LP**	66.66%	**MP**	88.88%	**HP**
**S32**	***Colletotrichum* sp.**	22.22%	**NP**	55.55%	**LP**	66.66%	**MP**
**Kishtwar**							
**K1**	***Fusarium oxysporum***	33.33%	**NP**	44.44%	**LP**	66.66%	**MP**
**K3**	***Fusarium solani***	0%	**NP**	22.22%	**NP**	55.55%	**LP**
**K4**	***Gibberella moniliformis***	0%	**NP**	0%	**NP**	11.11%	**NP**
**K5**	***Galactomyces* sp.**	0%	**NP**	11.11%	**NP**	44.44%	**LP**
**K8**	***Geotrichum candidum***	11.11%	**NP**	33.33%	**NP**	44.44%	**LP**
**K9**	***Geotrichum candidum***	0%	**NP**	11.11%	**NP**	55.55%	**LP**
**K11**	***Mucor circinelloides***	11.11%	**NP**	11.11%	**NP**	55.55%	**LP**
**K12**	***Galactomyces candidum***	0%	**NP**	11.11%	**NP**	33.33%	**NP**
**K13**	***Fusarium fujikuroi***	0%	**NP**	22.22%	**NP**	66.66%	**MP**
**K15**	***Geotrichum candidum***	11.11%	**NP**	11.11%	**NP**	22.22%	**NP**
**K16**	***Aspergillus flavus***	11.11%	**NP**	44.44%	**LP**	55.55%	**LP**
**K17**	***Aspergillus flavus***	11.11%	**NP**	44.44%	**LP**	66.66%	**MP**
**K20**	***Geotrichum candidum***	0%	**NP**	0%	**NP**	22.22%	**NP**
**K21**	***Fusarium solani***	0%	**NP**	44.44%	**LP**	66.66%	**MP**
**Ramban**							
**R1**	***Geotrichum candidum***	22.22%	**NP**	44.44%	**LP**	55.55%	**LP**
**R7**	***Galactomyces* sp.**	22.22%	**NP**	33.33%	**NP**	44.44%	**LP**
**R8**	***Fusarium* sp.**	33.33%	**NP**	55.55%	**LP**	66.66%	**MP**
**R9**	***Fusarium solani***	33.33%	**NP**	66.66%	**MP**	88.88%	**HP**
**R10**	***Galactomyces geotrichum***	22.22%	**NP**	44.44%	**LP**	55.55%	**LP**
**R11**	***Fusarium solani***	33.33%	**NP**	66.66%	**MP**	77.77%	**MP**
**R13**	***Mucor circinelloides***	22.22%	**NP**	33.33%	**NP**	33.33%	**NP**
**R14**	***Mucor circinelloides***	22.22%	**NP**	44.44%	**LP**	44.44%	**LP**
**R15**	***Mucor circinelloides***	33.33%	**NP**	55.55%	**LP**	66.66%	**MP**
**R16**	***Fusarium neocosmosporiellum***	44.44%	**LP**	77.77%	**MP**	77.77%	**MP**
**R17**	***Fusarium solani***	33.33%	**NP**	55.55%	**LP**	88.88%	**HP**
**R18**	***Fusarium solani***	33.33%	**NP**	66.66%	**MP**	88.88%	**HP**
**R19**	***Fusarium solani***	33.33%	**NP**	66.66%	**MP**	88.88%	**HP**
**R20**	***Mucor circinelloides***	22.22%	**NP**	44.44%	**LP**	55.55%	**LP**
**Fox R1 Accession No. KF663598 (Positive Control)**	***Fusarium oxysporum***	33.33%	**NP**	55.55%	**LP**	88.88%	**HP**

* NP- Non-pathogenic (below 40% disease severity); LP- Low-pathogenic (41-60% disease severity); MP- Moderately-pathogenic (61-80% disease severity) and HP-Highly-pathogenic (81-100% disease severity); **Fungal species name are represented in the same order as fungal isolate name.

## 4 Discussion

Previously, pathogenic fungal diversity associated with the saffron plant has been investigated on saffron corm samples including infected corms, corms that could or could not germinate, and corms that could germinate but were unable to bloom by both culture-dependent and -independent techniques (Gupta and Vakhlu, 2015; Ambardar et al., 2016; Bhagat et al., 2021; Shuwen et al., 2021; Mirghasempour et al., 2022b). Apart from the saffron corms, fungal diversity has also been estimated from roots, stems, leaves, and other tissues of saffron (El Aymani et al., 2019; Belfiori et al., 2021). As far as fungal diversity in the saffron fields is conserved, there are only two reports to our knowledge and both are from Morocco (Chamkhi et al., 2018; El Aymani et al., 2019). Therefore, the present study was initiated with an aim to unravel cultivable fungal diversity associated with saffron fields in Jammu and Kashmir in both traditional and non-traditional areas of saffron cultivation, i.e., traditional: Srinagar and Kishtwar, and non-traditional: Ramban. Furthermore, these fungal species were screened against saffron plant for causing symptoms of corm and root rot so as to evaluate the pathogenic and non-pathogenic fungi. Additionally the status of *F. oxysporum* as the most severe pathogen in saffron was also checked.

A total of 45 fungi were isolated from the saffron field soil of all the three regions. Ascomycota was the dominant community observed in the present study. Egidi et al. (2019) have also reported Ascomycota as the dominant fungal community in soil worldwide. Kumar et al. (2022b) reported the positive correlation of Ascomycota with phosphorus (P). They had investigated the effect of soil physical–chemical properties on bacterial and fungal community in two different compartments of soil of two different crop types—Chinese cabbage and flower cabbage. The soil with a high concentration of P has abundant Ascomycota that plays an important role in the absorption and utilization of soil P by the plants. Zhang et al. (2019) suggested the negative correlation of total nitrogen with the abundance of Mucoromycota.

The present study wherein samples have been evaluated in the vegetative phase is consistent with the earlier report from saffron cormosphere during the vegetative phase (Bhagat et al., 2021) but in contrast to the flowering and dominant phase (Ambardar et al., 2016). This confirms our previous hypothesis wherein we have reported that the mycobiome of cormosphere is dynamic and changes at each growth stage (Ambardar et al., 2016). Interestingly, studies on endophytic fungi of the saffron plant also report Ascomycota as a dominant endophytic fungal group (Wani et al., 2016; Belfiori et al., 2021; Jan et al., 2022). Ambardar et al. (2016) found Ascomycota to be dominant phylum in the bulk soil of saffron at both growth stages, i.e., dominant and flowering.

Seven genera, representing 45 OTUs in the present study, include *Fusarium*, *Geotrichum*, *Mucor*, *Aspergillus*, *Trichoderma*, *Galactomyces*, and *Colletotrichum*. The present report is in tune with the study conducted by El Aymani et al. (2019) on soil, roots, and corms of the saffron plant in the Taliouine region, Morocco. They found *Fusarium*, *Aspergillus*, *Trichoderma*, *Rhizopus*, and *Penicillium* genera in abundance. Interestingly, *Fusarium*, *Mucor*, and *Trichoderma* were the common genera observed in the present work and by Belfiori and colleagues in 10 different Italian saffron cultivation sites (Belfiori et al., 2021). *Trichoderma*, naturally in association with the saffron plant, has been reported by other research groups as well (El Aymani et al., 2019; Belfiori et al., 2021). Other genera that have been isolated by Belfiori et al. (2021) from the rotten saffron plant parts (corms, stems, and leaves) were *Rhizopus*, *Epicoccum, Aureobasidium*, and *Talaromyces*. In contrast to our results, Shuwen et al. (2021) have reported distinct fungal genera from corm samples of South Tai Lake Agricultural Park (Huzhou city, China) having different corm growth parameters such as germination and blooming. Infected corms had high levels of *Trichocomaceae* and *Talaromyces* genus, but non-germinating corms had high levels of the *Aspergillaceae* genus. In corms that could germinate but not bloom and in corms that could germinate and produce two blooms per corm, *Penicillium* and *Dothideomycetes* were found. *Cladosporium, Lambertella, Passalora, Eurotiomycetes, Eurotiales, Aspergillus, Cladosporiaceae*, and *Capnodiales* were the other fungal communities reported at the genus level by Shuwen et al. (2021).

Since few taxa and mostly *F. oxysporum* are associated in causing corm rot disease in saffron (Palmero et al., 2014; Gupta et al., 2020; Mirghasempour et al., 2022a), the isolated fungi in the present study were tested for pathogenicity to screen for pathogenic and non-pathogenic taxa. In our experiments with the previously isolated *F. oxysporum* R1 (Gupta and Vakhlu, 2015), it was established that it needs injury to invade the plant, and without injury, it cannot cause infection in the laboratory experiments (Bhagat et al., 2022). On similar lines, the pathogenicity was checked by injuring the corm as explained in detail in the *Materials and methods* section. According to the current findings, all the fungal species were pathogenic to corm and root but all fungi could cause disease only if the corm or root tissue was injured and no symptoms were observed in tissue without injury (**Figure 8**). In the present study, injury was found to be a must in the case of all the fungi screened as in the control, where the corms were sown in the soil infested with fungal spores but not injured. Since, in the present study, near-natural conditions were created by inoculating soil with fungal spores and planting corms and letting them grown naturally, we hypothesize that, in natural conditions, injury/natural wounds are the primary reason for fungal infection. There are reported reasons responsible for the occurrence of natural injury in corm such as injury being caused by the digging, storage, and re-sowing of corms (Gupta et al., 2021). In addition, nematodes and rodents have also been reported to attack the corms and cause injury (Khan and Sharma, 2020). Some researchers have reported the infection without injury and by just dipping the corm or germinated roots in the spore suspension (Palmero et al., 2014; Gupta et al., 2020; Mirghasempour et al., 2022b) but the present study and others have reported the infection after injuring the corm artificially (Gupta and Vakhlu, 2015; Wani et al., 2016; Wani et al., 2018; Zhang et al., 2020; Bhagat et al., 2022; Luo et al., 2022). There are many reports on other plants wherein the screening for pathogenicity is done by injury such as cork oak (Luque et al., 2000), kiwi fruit (Di Marco et al., 2004), grape vines (Halleen et al., 2007), *Dendrobium nobile*, and *Dendrobium officinale* (Sarsaiya et al., 2020), potato (Azil et al., 2021).

The establishment of Koch’s postulates suggested that *F. oxysporum*, *F. solani*, *F. fujikuroi*, *F. neocosmosporiellum*, and *G. moniliformis* (*Fusarium moniliformae*) were pathogenic to saffron but *F. oxysporum* was not the most severe. However, there was noticeable difference in the disease severity caused by various pathogens. *F. oxysporum* S12, *F. oxysporum* S30, and *F. oxysporum* K1 caused maximum disease severity of 75% at 30 dpi in saffron corms. The 75% disease severity was observed in other isolated strains such as *F. solani* K3, *G. moniliformis* K4, *G. candidum* K8, *M. circinelloides* K11, and *A. flavus* K16. *F. oxysporum formae speciales* such as *iridiacearum*, *gladioli*, and *saffrani* have already been recognized as the worst pathogens for saffron (Palmero et al., 2014; Najari et al., 2018; Belfiori et al., 2021; Gupta et al., 2021; Mirghasempour et al., 2022a). They cause corm rot disease in saffron and, therefore, resulted in severe losses in the saffron yield globally (Mirghasempour et al., 2022a). Interestingly, Wani et al. (2016) reported that endophytic *F. oxysporum* CSE15 exhibits latent pathogenic behavior in saffron and has strong antimycotic and plant growth-promoting properties. Moreover, the saprotroph, pathotroph, and symbiotroph trophic modes have all been well documented in the case of *Fusarium* genera (Belfiori et al., 2021). El Aymani et al. (2019) have presented *F. solani* as saffron-associated species. Additionally, *F. solani* can cause diseases in several crops including pea, *Zea mays* (Okello et al., 2019; Gibert et al., 2022). Quite a number of reports suggest that *F. oxysporum* is the most common cause of corm rot and devastating fungi (Yamamoto et al., 1954; Di Primo and Cappelli, 2000; Gupta et al., 2011; Gupta and Vakhlu, 2015; Gupta et al., 2020; Ahmad et al., 2022). The literature survey revealed *F. oxysporum* as a common corm rot pathogen, and to our understanding, the basis of this is the isolation of the *F. oxysporum* from the rotten corm, but there are reports that confirmed other fungal pathogens such as *Fusarium nirenbergiae*, *F. annulatum*, *F. commune, F. culmorum*, *F. roseum*, *Penicillium cyclopium*, *Aspergillus niger*, *Rhizopus oryzae*, and *Phoma* spp. as saffron corm rot agents (Gupta et al., 2021; Mirghasempour et al., 2022b). In addition, the reports claiming *F. oxysporum* to be the dominant corm rot causing pathogen are about two decades (and more) old, and no recent survey of saffron fields to find dominant fungal pathogen has been carried out (**Table 6**).

**Table 6:** Worldwide reports of saffron diseases caused by various pathogens, with the year of identification and source of isolation.

S. no.	Country	Diseases	Pathogen	Source	Year of identification of disease	References
1	Southern France	Violet root rot	*Rhizoctonia crocorum*	–	1728	Zadoks, 1981
2	Japan	Bacterial rot	*Bacillus croci*	–	1909	Mizusawa, 1923
		Dry rot	*Fusarium bulbigenum*	–	1933	Abe, 1933
		Corm rot	*Fusarium oxysporum* f. sp. *gladioli* and *Sclerotinia gladioli*	–	1954	Yamamoto et al., 1954
3	Italy	Corm rot	*Penicillium cyclopium* Westling	–	1973	Francesconi, 1973
		Corm rot	*Penicillium corymbiferum*	–	1991	Cappelli et al., 1991
		*Fusarium* corm rot	*Fusarium oxysporum* f. sp. *gladioli*	Infected saffron crops	2000	Di Primo and Cappelli, 2000
		Bacterial rot of saffron	*Burkholderia* isolates	–	2011	Fiori et al., 2011
		Corm rot	*Fusarium oxysporum*, *Trichoderma* sp., *Rhizopus oryzae*, *Mucor circinelloides*, *Epicoccum nigrum*, *Aureobasidium pullulans*, *Talaromyces pinophilus*	Rotten saffron plants	2021	Belfiori et al., 2021
4	Netherlands	Root rot	*Stromatinia gladioli*	–	1970	Schenk, 1970
5	Scotland	Corm rot	*Penicilium corymbiferum*	–	1985	Sutton and Wale, 1985
6	Greece	Corm rot	*Rhizoctonia crocorum*	–	1999	Goliaris, 1999
7	Morocco	Corm rot	*Fusarium oxysporum, Fusarium solani*, *F*. *culmorum*, *F*. *roseum*, *Fusarium* sp., *Aspergillus fumigatus*, *A*. *niger*, *Trichoderma* sp., *Penicillium* sp., and *Rhizopus oryzae*	Soils, corms and roots of saffron plants	2019	El Aymani et al., 2019
8	Iran	Wild saffron corm rot	*Fusarium oxysporum*	Rotten saffron corms	2018	Najari et al., 2018
9	China	Corm rot	*Pseudomonas gladioli*	–	1990	Xu and Ge, 1990
		Corm rot	*Trichocomaceae*, *Talaromyces*, *Eurotiomycetes*, *Eurotiales*, *Aspergillaceae*, *Aspergillus*, *Cladosporiaceae*, *Capnodiales*, *Penicillium*, *Cladosporium*, *Lambertella*, *Passalora*, and *Dothideomycetes*	Saffron corm	2021	Shuwen et al., 2021
			*Ilyonectria macrodidyma, Mortierella humili, Chaetomiaceae*, *Aspergillus*, *Saccharomycetales*, *Nectriaceae*, *Penicillium*, *Serratia*, and *Penicillium chrysogenum*	Soil and saffron corms	2019	Shuwen et al., 2019
		Corm rot	*Fusarium nirenbergiae*	Saffron corm	2022	Mirghasempour et al., 2022a
		Corm rot	*Fusarium* species	Rotten saffron corm	2022	Mirghasempour et al., 2022b
		Corm rot	*Penicillium* species	Diseased corms	2020	Zhang et al., 2020
10	(Kishtwar) India	Corm rot	*Macrophomina phaseolina*	–	1992	Thakur et al., 1992
	(Kashmir) India	Corm rot	*Fusarium*, *Rhizoctonia* and *Penicillium* species	–	2003	Hassan and Devi, 2003
	(Kishtwar) (India)	Sclerotial rot	*Sclerotium rolfsii*	Rotten corms	2007	Kalha et al., 2007
	Kashmir (India)	Corm rot	*Fusarium oxysporum* R1, *Fusarium saloni*, *Penicillium* species	Rotten saffron corm	2015	Gupta and Vakhlu, 2015
	14 sites of J&K (India)	Corm rot	*Alternaria alternata* CSE18, *Aspergillus pseudodeflectus* CSE36, *Penicillium pinophilum* CSE20, *Fusarium oxysporum* CSE15, *Penicillium canescens* CS3E4, *Talaromyces cellulolyticus* CS3E6, *Acremonium alternatum* CSF4	Saffron corms	2016	Wani et al., 2016
11	Spain	Corm rot	*Fusarium* species	Rotten corms	2014	Palmero et al., 2014
		Neck and corm rot	*Stromatinia gladioli*	**Asymptomatic corms**	2020	Muñoz et al., 2020
12	Srinagar (India)	Corm rot and root rot	*Fusarium oxysporum*, *Fusarium solani*, *Trichoderma* sp., *Trichoderma harzianum*, *Colletotrichum* sp.	Soil	**Present study**	
	Kishtwar (India)	Corm rot and root rot	*Fusarium oxysporum*, *Fusarium solani*, *Fusarium fujikuroi*, *Aspergillus flavus*, *Geotrichum candidum*, *Galactomyces candidum*, *Galactomyces* sp., *Mucor circinelloides*, *Gibberella moniliformis*	Soil		
	Ramban (India)	Corm rot and root rot	*Fusarium solani*, *Fusarium neocosmosporiellum*, *Fusarium* sp., *Geotrichum candidum*, *Galactomyces geotrichum*, *Galactomyces* sp., *Mucor circinelloides*	Soil		

To the best of our knowledge, *F. fujikuroi*, *F. neocosmosporiellum*, and *G. moniliformis* are being reported as pathogens from the saffron fields of Jammu and Kashmir, for the first time. The presence of these species has been studied in different plants. *F. fujikuroi* has previously been accounted as endophytic fungi, isolated from *Debregeasia salicifolia* (Nisa et al., 2020). Moreover, *F. fujikuroi* has been reported as herbicidal (Daniel et al., 2018). Nonetheless, *F. neocosmosporiellum* has earlier been reported as a potent pathogen of mango, causing mango malformation disease (Molina-Cárdenas et al., 2021). Additionally, *F. neocosmosporiellum* has also been isolated from the infected peanut plants (Xu et al., 2021). On the other hand, *G. moniliformis* is the well-known causal agent of ear rot and stalk rot diseases of maize (Gai et al., 2018).

*Colletotrichum* also has been documented as pathogens by many reports (Zakaria, 2021) such as sugarcane (Franco et al., 2022), capsicum (Giacomin et al., 2021), and banana (Sudarma et al., 2021). Moreover, *Colletotrichum* species have been reported as endophytes, having various bioactive metabolites (Santra and Banerjee, 2022). The *Aspergillus* species isolated in this study on close examination resembled *A. flavus*; however, El Aymani et al. (2019) have found *Aspergillus fumigates* and *A*. *niger* species on close association with the saffron plant. In addition to all the above-mentioned fungal species, one more species, *G. candidum*, whose sexual stage is named *G. geotrichum*, was isolated in the present study. The asexual stage *G. candidum* belongs to the family Moniliaceae, whereas the sexual stage *G. geotrichum* belongs to the Candida family. There are more than 10 synonyms reported for *G. candidum* (Ma et al., 2018). In the present study, both the sexual and the asexual stage were found and both were pathogenic to the saffron plant. As such, there are no data available regarding *G. candidum* in context to the saffron plant. However, previous studies have already revealed *G. candidum* as a strongly virulent pathogen to carrots (Horita and Hatta, 2016), citrus plants (Wang et al., 2021), strawberry (Ma et al., 2018), and tomatoes (Thomidis et al., 2021).

In accordance with our studies, *M. circinelloides* and *Trichoderma* sp. have also been isolated from the rotten saffron plant parts by Belfiori et al. (2021). Although *M. circinelloides* is a well-known phytopathogen (Medina-Córdova et al., 2016), members of the *Trichoderma* genus are popular for their protection activities and represent global distribution as plant endophytes. Moreover, they are well-known antagonists for pathogenic fungal species and also act as plant growth promoters (Cuervo-Parra et al., 2022). *Trichoderma* have been used to manage many diseases and hence are commercially available biological control agents. They have been applied to a variety of plants for disease management, including tomato (Li et al., 2018), soybeans (Cruz et al., 2017), beans (Sánchez-García et al., 2017), cocoa (Romero-Cortes et al., 2019), and onion (Bunbury-Blanchette and Walker, 2019). In the saffron plant, Gupta et al. (2020) have evaluated the efficacy of *Trichoderma asperellum* on the corm rot causative agent, *F. oxysporum*, and found reduction in the disease incidence. The isolated *T. harzianum* has been observed as a pathogen in our study; however, it was an evident biocontrol agent reported by other studies (da Silva et al., 2022; Moreira et al., 2022). Hence, further research is required to determine its potential role as a pathogen antagonist.

Out of the fungi isolated, we found all of them to be pathogenic with varying degrees of disease severity. The six *F. oxysporum* strains isolated in the present study are not exclusively severe saffron corm rot pathogens; there were other fungi isolated that were equally severe such as *F. solani, G. moniliformis, G. candidum, M. circinelloides*, and *A. flavus*. However, out of six isolated *F. oxysporum* strains, none was found to be the most severe saffron root rot pathogen. In addition, we found that it is the injury that makes corm susceptible to the opportunistic fungi in the soil, and all the fungi that had been isolated were pathogenic only if a corm/root was injured, not otherwise. Under natural conditions, however, other parameters may play a role in infection such as humidity and temperature (Victorino et al., 2021), but to our understanding, injury is most fatal if it is caused by natural conditions such as farming practices, rodents, and nematodes.

## 5 Conclusion

Pathogens are evolving fast, invading new plant hosts, and climate change is adding to that. Based on decades-old reports, *F. oxysporum* f. sp. *gladiolus* has been reported as the most severe pathogen of saffron, causing corm rot without any molecular phylogeny. There are various strains of *F. oxysporum* present in the soil as it is the dominant taxa of the most dominant soil phylum, i.e., Ascomycota. The severity in the strains may vary, and this needs to be validated by data. The data for the isolation and characterization of the pathogenic *F. oxysporum* causing corm rot are isolated, random, and scarce. In our opinion, to establish the pathogen as most severe, causing the devastating rot to the world’s costliest spice, comparative data with robust experimentation are required. Moreover, in the present study, it was also observed that all the fungal isolates cause rot symptoms, when the corm is injured; thus, it is injury that can be a major cause of the corm rot and yield loss rather than the pathogen. Although this hypothesis needs to be further validated in field experiments, we strongly recommend sowing injury-free corms so as to prevent future potential outbreaks instead of the unchecked use of chemical fungicides. This study revealed that Srinagar, which is a traditional area for saffron cultivation in India, has less diverse fungal species as compared to the other two studied locations and that *T. harzianum*, which is used as a biocontrol, is also a potential pathogen. We recommend screening of symptoms and injury-free quality corms before sowing and regulated use of chemical as well as biological agents in saffron fields for sustainable saffron production.

## Data availability statement

The datasets presented in this study can be found in online repositories. The names of the repository/repositories and accession number(s) can be found in the article/**Supplementary Material**.

## Author contributions

JV conceived the research idea. RM and JV designed the experiments. RM with the help of TA performed the experiments. JV supervised the experiments. RM performed data analysis. JV and RM together drafted the manuscript. NB provided inputs in manuscript. All authors contributed to the article and approved the submitted version.
