# Supplementary material for: Injury and not the pathogen is the primary cause of corm rot in Crocus sativus (saffron)
Source: Front Plant Sci. 2023 Jan 24;14:1074185. doi: 10.3389/fpls.2023.1074185 (PMC9902776; doi:10.3389/fpls.2023.1074185)
Supplement: Supplementary file 1 [file DataSheet_1.docx]

**
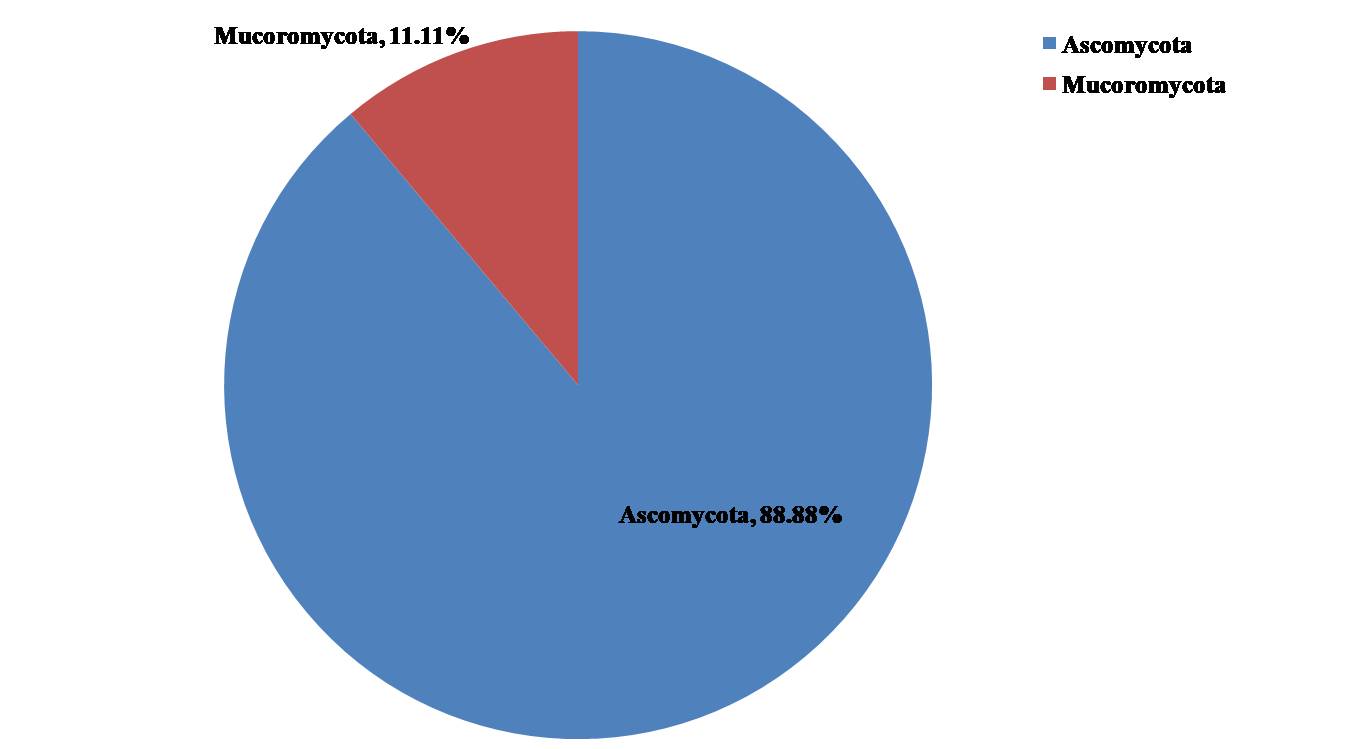
**

**Supplementary figure 1:** Pie chart representing the frequencies of operational taxanomic units (OTUs) belonging to each phylum


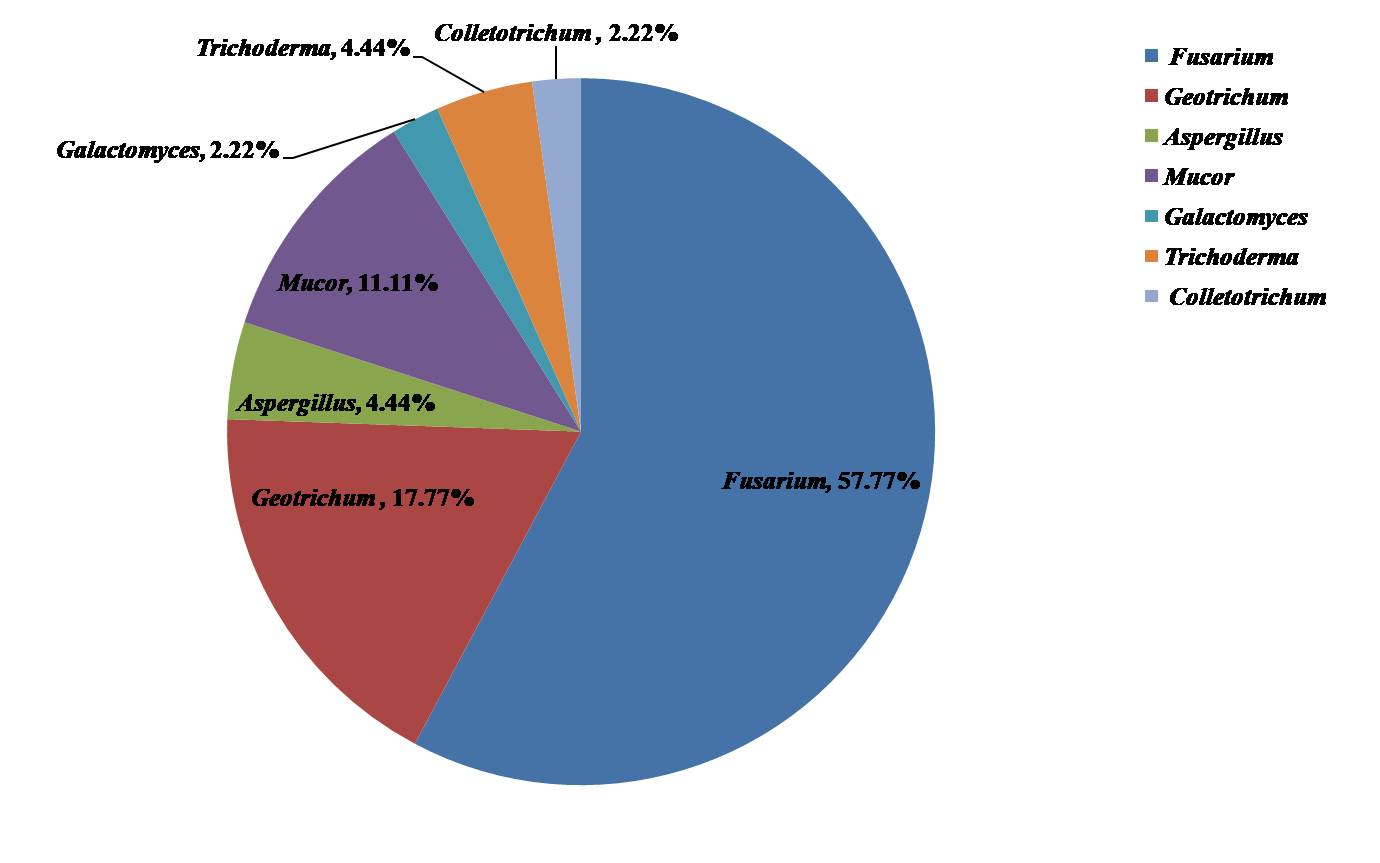


**Supplementary figure 2:** Pie chart representing the frequencies of operational taxanomic units (OTUs) belonging to each genus
